# Perspectives of health educators and interviewers in a randomized controlled trial of a postpartum diabetes prevention program for Latinas: a qualitative assessment

**DOI:** 10.1186/s12913-019-4207-x

**Published:** 2019-06-06

**Authors:** Aline Gubrium, Denise Leckenby, Megan Ward Harvey, Bess H. Marcus, Milagros C. Rosal, Lisa Chasan-Taber

**Affiliations:** 1 Department of Health Promotion and Policy, School of Public Health & Health Sciences, University of Massachusetts, Amherst, MA USA; 2 Department of Biostatistics & Epidemiology, School of Public Health & Health Sciences, 401 Arnold House, University of Massachusetts, 715 North Pleasant Street, Amherst, MA 01003-9304 USA; 3 Department of Behavioral and Social Sciences, Brown University School of Public Health, Providence, Rhode Island USA; 4 Division of Preventive and Behavioral Medicine, Department of Medicine, University of Massachusetts Medical School, Worcester, MA USA

**Keywords:** Process evaluation, Researchers’ perspective, Hispanic, Postpartum, Qualitative, Lifestyle intervention, Randomised controlled trial, Latina

## Abstract

**Background:**

Lifestyle interventions regularly rely on study staff to implement the intervention and collect outcomes data directly from study participants. This study describes the experiences of project staff in two randomized controlled trials of a postpartum lifestyle intervention to reduce risk factors for type 2 diabetes in Latinas. Latinas are the fastest growing minority group in the U.S. and have the highest rates of type 2 diabetes after a diagnosis of gestational diabetes mellitus. The challenges of implementing lifestyle interventions for postpartum women have been poorly documented.

**Methods:**

A qualitative focus group was conducted with eight staff members (five health educators and three health interviewers) involved in *Proyecto Mamá* and *Estudio Parto*. The discussion was audio recorded, transcribed, and coded in NVivo. Focus group topics included: 1) participant recruitment, 2) participant retention, 3) implementation of the lifestyle intervention, 4) assessment of behavior change, 5) overall challenges and rewarding aspects of the trial, and 6) recommended changes for future trials.

**Results:**

Key themes emerged regarding enabling factors and barriers to implementing a lifestyle intervention in postpartum Latinas. Enabling factors included: a) the staff’s belief in the importance of the intervention, b) opportunities associated with the longitudinal nature of the trial, c) belief that the staff could empower participants to make behavior change, d) benefits of flexible intervention sessions, and e) connection with participants due to shared cultural backgrounds. Barriers included: a) participant stressors: home, food, and financial insecurity, b) low health literacy, c) issues related to recent immigration to the continental U.S., d) handling participant resistance to behavior change, e) involvement of family members in assessment visits, f) limitations of the assessment tools, and g) time limitations.

**Conclusions:**

Findings highlight the challenging contexts that many study participants face, and shed light on the potentially influential role of health educators and interviewers in intervention implementation and data collection. Specific recommendations are made for strategies to improve adherence to diabetes prevention programs in postpartum underserved and minority populations in this challenging, transitional period of life.

**Trial registration:**

NCT01679210. Registered 5 September 2012; NCT01868230. Registered 4 June 2013.

## Background

The postpartum period is a critical time-period for interventions designed to prevent subsequent onset of diabetes in vulnerable populations. Worldwide, the number of people with diabetes is projected to rise from 382 million in 2013 to 592 million in 2030 [1]. At the same time, the age at onset for type 2 diabetes is decreasing, highlighting the importance of identifying high-risk groups early in order to implement prevention efforts [2]. One such high-risk group is women who develop gestational diabetes mellitus (GDM) or more mild forms of glucose intolerance in pregnancy [3, 4]. Women who develop GDM in pregnancy have a 7-fold higher risk for future type 2 diabetes [4]. Indeed, women with recent gestational glucose intolerance are at increased risk of progression to prediabetes or diabetes as early as one year postpartum [5].

Latinas are the fastest growing minority group in the U.S. [6] and are disproportionately affected by overweight and obesity, excessive gestational weight gain, and GDM [7]. In addition, as compared to non-Latinas, Latinas with a history of GDM are less aware of diabetes risk factors and prevention strategies, such as physical activity, dietary behaviors, and weight management [8]. In spite of these observations, due to socioeconomic circumstances, differences in educational background, health literacy, and language barriers, Latinas have had limited access to interventions that promote healthy lifestyles [9].

Systematic reviews and meta-analyses have found that postpartum lifestyle interventions have the potential to lead to clinically significant weight reduction, improvements in physical activity, dietary behaviors, and other diabetes risk factors [10–13]. However the number of postpartum interventions has been small, with widely diverse content and varying levels of participant adherence, and therefore their feasibility remains unclear. For example, poor retention rates have been reported in postpartum trials of exercise for weight management, with dropout rates ranging from 17 to 40% [11]. In addition, the value of lifestyle interventions for women of different ethnic backgrounds remains unclear [10, 11].

Lifestyle interventions regularly rely on study staff to implement the intervention and collect outcomes data directly from study participants. To date, little has been done to identify the individual components of lifestyle interventions, and the aspects of their implementation, that are critical to success [14–16]. Qualitative research tools can identify staff persons’ experiences and knowledge that might otherwise remain hidden through more traditional quantitative measures [17, 18]. These staff have a particularly intimate view of the lives of study participants, and may be uniquely qualified to identify factors that influence the ultimate success of an intervention.

To our knowledge, no studies to date have collected information on the experiences of health educators and interviewers involved in implementing a lifestyle intervention to postpartum women. Prior studies implemented during pregnancy have described the perspectives of health educators and interviewers in a randomized trial of prenatal smoking cessation [14], the perspectives of interviewers in a case-control study of preterm birth [15], and the perspectives of interviewers in the Danish National Birth Cohort Study [16].

Therefore, we present results from a focus group held with a team of health educators and interviewers who worked on two postpartum randomized trials of diabetes prevention programs for Latinas: *Estudio PARTO* (Project Aiming to Reduce Type twO diabetes) and *Proyecto Mamá*. Our goal was to qualitatively assess the perspectives of these staff to inform future postpartum diabetes prevention programs.

## Methods

### Proyecto Mamá and Estudio PARTO

The trial protocols for Proyecto Mamá [19] and Estudio PARTO [9] have been previously published. Both trials were based at the ambulatory obstetrical practices of Baystate Medical Center in Western Massachusetts and were ongoing at the time of the focus groups.

*Proyecto Mamá* was a randomized controlled trial conducted from June 2014 to July 2018 to test the efficacy of a culturally and linguistically modified, individually-tailored lifestyle intervention to reduce excess gestational weight gain, increase postpartum weight loss, and improve maternal metabolic status among overweight/obese Latinas. Eligible women were recruited in early pregnancy and randomly assigned to a Lifestyle Intervention (*n* = 150) or a Comparison Health and Wellness (control) intervention (*n* = 150). Randomization was stratified based on age (< 30, > 30 years) and prepregnancy BMI (overweight > 25- < 30 kg/m^2^ vs. obese > 30 kg/m^2^). Within each stratum, a blocked randomization was used such that both treatment groups were assigned an equal number of times in each set of four sequentially enrolled patients.

*Estudio PARTO* was a randomized controlled trial conducted from January 2013 to December 2017 to test the efficacy of a culturally and linguistically modified, individually-tailored lifestyle intervention to reduce risk factors for type 2 diabetes and cardiovascular disease among postpartum Latinas with a history of abnormal glucose tolerance during pregnancy. Eligible women were randomly assigned to a Lifestyle Intervention (*n* = 150) or a Health & Wellness (control) Intervention (*n* = 150). Randomization occurred after the diagnosis of GDM (24–28 weeks gestation) and after completion of the baseline assessment. Randomization was stratified based on study site and the results of the diagnostic GDM screen (one vs. at least two glucose values during the diagnostic test meeting or exceeding thresholds).

For both trials, women were informed of the aims and procedures of the project, and eligible and interested women were consented in writing for participation in the study. Both studies were approved by the Institutional Review Boards of the University of Massachusetts-Amherst and Baystate Health.

Multimodal contacts (i.e., in-person, telephone, and mailed materials) were used to deliver the intervention from pregnancy (preparatory phase) through 12 months postpartum. This high-reach, low-cost strategy was selected such that findings could readily be translated into clinical practice in underserved and minority populations.

The interventions were delivered by bicultural and bilingual trained health educators. The lifestyle interventions utilized culturally and linguistically modified, motivationally targeted, individually tailored intervention materials. The lifestyle interventions were adapted from evidence-based approaches promulgated by the Institute of Medicine [7], American College of Obstetrician and Gynecologists [20]; and the American Diabetes Association [21]. Specifically, the targets of the intervention were to achieve Institute of Medicine guidelines for gestational weight gain and postpartum weight loss [7]; American Congress of Obstetrician and Gynecologist guidelines for postpartum physical activity [20]; and American Diabetes Association guidelines for diet [21]. The interventions drew from Social Cognitive Theory [22] and the Transtheoretical Model [23] and took into account the specific social, cultural, economic, and environmental challenges faced by Latinas [8, 24, 25].

Assessments were conducted during pregnancy, and at 6-weeks, 6-months, and 12-months postpartum by trained bicultural and bilingual health interviewers blinded to the intervention arm. Measures included weight, physical activity assessed via the Pregnancy Physical Activity Questionnaire [26], and diet measured via three unannounced 24-h dietary recalls.

### Design

An open-ended qualitative focus group was led by an investigator (DL) not known to the health educators/interviewers and who was not involved with the trials. DL is a female doctoral student in Community Health Education with a Master’s degree in Public Health and more than three years of qualitative research experience in one-on-one and focus group interviewing. She moderated the sessions using a focus group discussion guide informed by prior qualitative work among research staff [14, 17] The focus group began with an introduction where DL described her background, interest in the topic, and reasons for conducting the research. Following this introduction, open-ended questions were used to understand the health educators’ and interviewers’ perspectives on implementing this lifestyle intervention. Topics discussed included: 1) participant recruitment, 2) participant retention, 3) implementation of the lifestyle intervention, 4) assessment of behavior change, 5) overall challenges and rewarding aspects of the trial, and 6) recommended changes for future trials.

The focus group was conducted in a conference room at the University of Massachusetts and was limited to the moderator and the study staff. The focus group lasted ninety minutes, and was audio recorded and transcribed verbatim. Field notes were not made and data saturation was not discussed. Major themes were derived from the data through a grounded theory driven content analysis of the transcript. [18]. A coding key was developed with definitions, descriptions, exemplary quotes organized underneath the major themes. Quotations were identified through an anonymous numbering system. Transcripts were not returned to participants for comment or correction. Through the thematic content analysis grounded theory [18], commonalities and distinctions amongst perceptions of the staff were explored, yielding interpretive and illustrative findings.

#### Focus group participants

The complete roster of eight staff members (five health educators and three health interviewers) were invited in person to participate in the focus group. One additional health interviewer who was currently working on the trial was invited to participate but was not available. Staff provided written informed consent before participating in the focus group. All of the study staff had worked on both studies for a range of 2 to 6 years. Seven of the eight staff members were bicultural; specifically of Puerto Rican, South American, Central American, and Cuban heritage. All of the staff were female.

Staff had been trained for their roles in Estudio Parto during a three-day course, including sessions on the study protocol, and participant tracking and retention. Health educators were further trained on recruitment, obtaining informed consent, randomization, and motivational interviewing. Health interviewers were further trained on objective data collection skills and physical measurements. For all staff, this training was followed by a one-week period spent on site shadowing current staff.

#### Data management and analysis

We used NVivo 11 to organize and code the transcript data (NVivo qualitative data analysis software; QSR International Pty Ltd. Version 11.4.1). DL conducted multiple listenings and subsequent readings of the text to generate a provisional coding framework for each segment of text, and in consultation with AG, built a coding framework. DL thematically coded the transcript independently and created new codes where these were required. Identified themes are illustrated by selected anonymized quotes, which are characteristic of the data. Focus group participants did not provide feedback on the findings.

## Results

The thematic analysis resulted in the following enabling factors and barriers to implementing a trial of a lifestyle intervention among postpartum Latinas.

### Enabling factors in implementing a trial

#### Belief in the importance of the intervention

The health educators believed in the importance of the content of the lifestyle intervention. They valued the opportunity to talk about healthy behaviors, such as increasing physical activity and improving diet quality. Health educators appreciated the chance to provide participants with a view of their behavior and help participants identify what they could change.

Reflecting on the importance of the intervention, one health educator highlighted the opportunity to provide critical information to participants:

*Having the opportunity to talk about what’s important. Physical activity. Or to eat healthy. It’s like a window. An opportunity for them to see what they are doing good at the moment or they can see what they can change. So, for me, that’s something that I really really like… It doesn’t matter if they make changes or not.*

#### Longitudinal nature of the trial

The health educators and interviewers had a favorable view of the longitudinal study design and the dedicated contact time for intervention delivery and assessment. They believed that contact time and follow-up were not only necessary to evaluate the impact of the intervention, but also essential to building a relationship with study participants. Without the opportunity for this connection to deepen over time, the health educators felt that the intervention would not have had as much impact.

One health educator noted the importance of extended contact with participants:

*I think one of the great things about the intervention is the length of the study. So, having that year to follow them after birth, that long trajectory. Longitudinal study makes all the difference in the world. If you’d just met them once or twice or a few times during the study. You’d never be able to see that change. Because for some of them there’s a lot of ups and downs and they lose 30 lbs., they gain 40, then their back down 10. They lose a job, they get a job. They move three times. They’re in a shelter. So, the longitudinal aspect of the study was crucial in being able to see what happens over time.*

Long-term relationships were viewed as building connections between project staff and study participants. The importance of relationship building and connection was a theme articulated by many of the focus group respondents. One health educator noted:

*There’s still a consistent sense of companionship. That rapport lets them share much more. Because, especially, initially, they stay superficial. A lot of them who are more defensive, stay at a very superficial level. And then, to get through those defense barriers and layers, and the fact that you’ve seen them through the harder points and you see, well that one’s had this problem with that boyfriend…and you know, you’ve been there the whole time. I feel like they share so much more.*

Another health interviewer also commented:

*And I do see that we are creating a relationship. Right? … And actually, I just conducted a couple of endings. One of them, she was saying, “and now you’re gone? Who is going to call me? If you have something else, please call me. Or just to say hello. Or just to say I miss you.”*

#### Empowering participants to make behavior change

Health educators participating in the focus group valued the opportunity to empower participants to make changes in their behaviors. They viewed themselves as advocates for health changes and, likewise, appreciated even incremental improvements in participant behavior.

*I mean the point of the intervention is not to change the whole habit. Like if we see that they stop drinking soda. That’s something that we are making progress on. Whenever she came she had changed. She had stopped drinking soda. She was drinking water. So, these women, they do. When they feel empowered. And they feel like they matter.*

Another health educator noted,

*It’s been interesting to see, from where they’ve started, to hear what they say they’ve achieved. Getting to that point where they see what they’re proudest of, what they say is most important about the study, what they say they learned and took away from the study. These might not even be things that I noted during my visits with them. So that’s really important.*

#### Flexible nature of the sessions

The health educators enjoyed the flexible nature of the intervention and, at the same time, saw it as critical for the effectiveness of the intervention. The use of motivational interviewing allowed the health educators to adapt the delivery of the intervention to the participants’ own motivations to make positive behavior changes and accomplish their self-identified goals.

One health educator reflected:

*As health educators, we still have a sort of loose structure that we follow, but it’s flexible and its conversational. And in that, it’s enriching for us as we are doing the job and it’s also, I think, you know, it’s where we are seeing the difference with [participants].*

#### Shared cultural backgrounds

The study staff viewed their common cultural experiences with study participants, such as shared language, food, and culture, as a positive aspect of the study. The staff felt that this shared background and the availability of Spanish-language tools engendered a cultural connectedness with participants that improved their effectiveness in delivering the intervention.

One health interviewer spoke of the importance of using a common language “from home” to connect with participants:

*And they hear you speaking and your accent, you’re from the island. They realize you are Puerto Rican and they get excited! And they’re like, “We’re both from the island.” And that’s the way we make that relationship.*

Another health educator commented on the importance of a shared sense of home and culture:

*..that relationship with somebody who knows what I’m going through: maybe being away from where you are from or being an immigrant here. Or however you want to describe yourself. I think that being able to be that for them, for some of them have just moved and they are just missing where they are from and having somebody who sort of shared the same life. … for them it’s kind of enriching I would say.*

A health interviewer noted that connections over cultural foods contributed to relationship building and staff members’ empathy with participants:

*When they call you, sometimes, they are actually aware that they are going to be held accountable for what they ate, so they’re like, “Oooo, I know I should not have had like four campurias,” which is like a fried thing that we have on the island. “But they were so good that I didn’t resist it. You know what I mean right?” And I’m like, “Yeah! I understand”.*

### Barriers to implementing a trial

#### Participant stressors: home, food, and financial insecurity

Study staff spoke of participants facing multiple life stressors. Participants were often single mothers, frequently moved residences or lived in shelters, faced food insecurity, and lacked social support. Participant contact information and telephone numbers changed often. Life stressors and logistical challenges faced by the participants made it challenging for study staff to maintain contact with them over the course of follow-up.

Several staff members were critical of a narrow focus on prescribed behavior in the intervention, feeling that, as designed, the intervention was not able to take into account broader structural issues faced by participants in their daily lives:

A health educator commented:

*…[the intervention] doesn’t take into account like food insecurity either. Like in a lot of these women, some of them really have to go to a food pantry to get food. So, we’re going to tell them, get some fresh food and some vegetables on your plate. But they don’t have the money or the access to buy that.*

A health interviewer noted:

*The type of life that they are having, like any of us, when you are so stressed out for things that happen in your daily life. It’s heavy. Or you don’t have an income coming. You have to worry about everything else because you are single with children. I think that for the mothers, when it gets to the point that everything that they do, they do it in a rush.*

Another health interviewer noted:

*Well the nature of their life, often makes it very difficult. So, I have some women and I’ve known them less than a year and they’ve had three or four different phone numbers or they’re not available past the first week of every month, because they’ve used all their minutes up. Or their kid has dropped their phone in the toilet. Whatever all these reasons, it makes the consistency throughout the study a difficult thing. So sometimes it takes months to find somebody. And it’s because of those reasons. It might be that they’re moving, everything’s fluid. Often times there are several different partners involved. There’s several different houses involved. I have couples living out of their cars. So that’s been a challenge working with this population.*

*There is a good 20% of them who are doing good. They are happy-go-lucky. They give you their time. But then you look at their background. They have a house. They have a partner. They haven’t changed phones. They haven’t gone to a shelter. So, everything is just more steady. They are fine. They function. But this is not the case for the majority.*

#### Health literacy

Staff also found it challenging to implement the intervention when participants did not have a basic understanding of healthy eating and exercise behaviors. In addition, cultural beliefs regarding eating, particularly the social nature of food preparation and eating, were sometimes at odds with the specific individual behaviors encouraged by the intervention.

A health interviewer commented:

*You have women who tend to eat a lot of starches. You have tortilla, you have white rice every day, you have five tortillas plus beans. The sense is that they don’t see the reality of what it is to be eating right. And I think that is one of the problems or something that the study lacks. There should be just a little space, to explain these concepts. Because [the] women don’t know these concepts.*

A health educator noted:

*You have the material. I mean it’s right there. But let’s be honest, how long does it take, I mean anybody, even us, who has the information about this. To actually say, “Okay, yes I need to do this change.” We’re conscious of all this because we’re academics and we know. So we go to the gym because we know it’s important. But these people don’t have that.*

*Sometimes they do know that it is not a good thing for their health. But sometimes it’s just that, “if I’m going to be cooking for my whole family, and I know that my kids are not going to eat that healthy food...” Because family values are so important around food.*

#### Recent immigration to the continental U.S.

Some of the study participants had recently immigrated to the continental U.S. and faced a unique set of challenges that study staff viewed as barriers to the effectiveness of the intervention. In addition, staff suggested that cultural tailoring might need to go deeper to acknowledge historical, political and cultural dimensions that inform meanings of food, nutrition, and exercise.

One health educator noted:

…*you have people who just moved here from Nicaragua, or Salvador or Honduras or Guatemala. And you cannot even put them in the same category as Puerto Ricans who have been living in Holyoke or Springfield or even the island… You know there is no reference, no cultural reference for exercise. Or for setting goals. You know? They walk because their neighbor is having a baby three miles away and they are going to her hut to help her give birth to the baby on the dirt floor.*

#### Participant resistance to behavior change

Along with their sensitivity to the daily struggles of many of the study participants, health educators wrestled with participant resistance to making healthy behavior changes, and at times the slow pace of change.

*Before I came here, I ate white rice every single day of my life. For lunch and dinner. And you have to have your white rice. And I’m not eating really good food. But you know, these concepts are difficult to grasp. So, I mean, not only to build that relationship with the participants, but also make sure that they are understanding the importance. Why it is important to change just a little bit.*

#### The presence of family members

Health educators and interviewers also highlighted challenges with having boyfriends, husbands, mothers or healthcare aides in the room with the participant during either the in-person or telephone sessions. Regardless of whether these other individuals were encouraging or critical, the study staff felt that their presence negatively impacted the integrity of participant responses, leading to either an over-reporting of socially desirable responses or an underreporting of behaviors at odds with cultural and/or gendered expectations. At times, others in the room would answer for the participants.

One health educator reflected:

*Like the questions, “Have you felt so sad? Have you had difficulty to fall asleep? Sometimes do you feel like crying?” And their husbands are there and you can tell from their face that, “Yeah, I cry every night.” But they tell you, “No, I feel fine” Just because their husband is there. Or their mother-in-law. And they don’t want them to know.*

Another health interviewer noted:

*There are the mothers or grandmothers. Or also with these aides. And I asked this participant, “Did you do this…or...?” And [mimicking a health aide’s voice], “No she didn’t!”*

#### Limitations of the assessment tools

Study staff also expressed some concerns about the lack of colloquial language used in the intervention and assessment tools, specifically in the quality of the Spanish translations.

One health interviewer noted:

*I mean obviously, these questionnaires are partially designed to be culturally relevant. So, there’s little things referenced about how Hispanics might do things that Americans don’t. In terms of eating, in terms of exercising. But the actual translation of the instruments …like it’s the way that you would speak if you were writing and it’s not the way that people talk to one another.*

#### Time limitations

Finally, study staff also expressed frustration with the time demands of the assessment tools.

A health educator noted:

*…one of our roles is acting as a sounding board, I think, for women. And unfortunately, we don’t get enough time to do that due to the length and the monotony of the questionnaire. Because, really, we would love to get a lot richer and probably a lot more useful material at the end of the day.*

## Discussion

Our qualitative study of the perceptions of study staff conducting a randomized trial of a diabetes prevention program among postpartum Latinas identified both enabling factors and challenging aspects. Enabling factors included: a) the staff’s belief in the importance of the intervention, b) opportunities associated with the longitudinal nature of the trial, c) belief that they could empower participants to make behavior change, d) benefits of flexible intervention sessions, and e) connection with participants due to shared cultural backgrounds. Barriers included: a) participant stressors: home, food, and financial insecurity, b) low health literacy, c) issues related to recent immigration to the continental U.S., d) handling participant resistance to behavior change, e) involvement of family members in assessment visits, f) limitations of the assessment tools, and g) time limitations.

Overall, the health educators and interviewers valued most the relationships they developed with study participants within the constraints of the research environment. The staff appreciated the insights into participant’s resilience, even in the face of the food, home, and financial insecurity faced by many. The overarching impression from staff was a sense of respect for the study participants. The rewarding aspect of these relationships was viewed, by the staff, as a counterbalance to the more challenging aspects of their roles.

The staff believed that their personal connections with participants was the greatest enabler of positive behavior change and participant retention. Other trials conducted in low-income and minority pregnant and postpartum populations have found that recruitment and retention rates are positively influenced by staff with training in patient-centered techniques grounded in a health equity framework, as well as a flexible protocol tailored to the unique needs of this population [27, 28]. Our health educators and interviewers shared cultural backgrounds with participants and were similar in age, which they reported as having enhanced the quality of their relationships with participants. Consistent with prior studies in pregnant and postpartum minority women, the staff felt that the flexible nature of the protocol and participant-centered approach of the intervention facilitated both positive behavior change and trial retention [27, 28].

An important finding of the focus group was the rich information on the stressors experienced by the participants. Staff insights into the extent of food and housing insecurity in participants’ lives would otherwise have been missed by the quantitative assessment tools used in the study. The staff’s perception that these factors were barriers to behavior change are consistent with prior reviews that found that financial, housing, and food insecurity negatively impact participant ability to fully participate in all components of the intervention [12]. A postpartum lifestyle intervention designed to address the needs of women who are underserved is complicated by challenges that underserved women face. In other words, those women who could benefit the most from a postpartum lifestyle intervention may have the least time, energy, and resources to do so.

Staff on this trial were highly adept at balancing research expectations and their relationships with participants. Staff were directed to follow a fairly demanding study protocol within relatively certain time constraints, while engaging in a personal manner with participants and developing a strong relationship with some of them. Prior findings from qualitative interviews among staff suggest that trials such as ours consider the emotional impact these dual responsibilities place on study staff and include regular efforts to support staff members (i.e., debriefing strategies) [17]. For example, study staff participated in monthly in-person staff meetings and weekly telephone meetings calls which included dedicated time for health educators and interviewers to discuss their ongoing relationships with study participants, ask each other for help, and openly process their experiences.

While the study staff appreciated the availability of intervention materials tailored to the Latina culture and language, they felt that these materials could be strengthened through further tailoring to specific Latina subgroups or through limiting the study to a single Latina subgroup. They also made specific suggestions for shifting the academic tone of the Spanish translations of the intervention and assessment materials to be more reflective of everyday, colloquial conversation. Overall, staff felt that shortening assessment tools, reducing their number, or converting some of the questions into open-ended responses would increase their time to implement the intervention and build relationships with study participants. Mixed-methods data collection strategies that prioritize process can be effective in informing intervention and health promotion [29].

Staff noted that participant perceptions of family and cultural expectations were an important factor influencing the participants’ ability to comply with their behavior change goals. Family values were most often raised in the context of decisions about food choices, with staff suggesting that family members be involved in deeper, more meaningful ways in the intervention. These suggestions are consistent with those of a systematic review of lifestyle interventions in overweight/obese pregnant and postpartum women, which call for the development of interventions that target partners and family members [10]. Partners, in particular, have been identified as important enablers of regular physical activity in childbearing and childrearing women [10]. Broadening the intervention to engage the wider family network surrounding pregnant and postpartum women would help to address staff concerns that recommended levels of physical activity, dietary guidelines, and weight management behaviors may be in conflict with the participants’ cultural and family expectations.

While this study provides the first insights from study staff conducting a trial of a postpartum lifestyle intervention, it also faces several limitations. For example, it cannot be determined if the staff members’ perspective on the importance of their relationship with participants was similarly valued by the participants themselves. We also cannot establish, from this data, whether the quality of the staff-participant relationship was associated with positive behavior change. Future planned analysis of participant satisfaction surveys administered at the end of participant follow-up in *Estudio PARTO* and *Proyecto Mamá* will help to address this question.

While the focus group moderator was unknown to the study staff, and not associated with the study, social desirability bias may have constrained negative feedback. However, the fact that the staff reported a number of challenges to implementing the intervention reduces this concern. In addition, the staff were used to routine debriefing meetings where study challenges were discussed which likely facilitated their comfort level in sharing their perspectives. As compared to individual one-on-one interviews, the focus group method had the advantage of enabling discussion from and between the staff members, rather than being directed by the moderator.

It is important to acknowledge that this trial was focused on pregnant and postpartum Latinas of primarily Puerto Rican background, the largest Latina subgroup in the Northeast [30]. Findings from this qualitative study may not be generalizable to other Latina subgroups as cultural differences in groups may be important to consider.

## Conclusions

With the growing rates of diabetes and obesity in U.S. women, efforts to improve the effectiveness of lifestyle interventions for the prevention of diabetes in high-risk women becomes critical. This qualitative study adds to the limited process data concerning the challenges of implementing trials of lifestyle interventions for Latina postpartum and underserved populations. Findings highlight the challenging contexts that many study participants face, and shed light on the potentially influential role of health educators and interviewers in intervention implementation and data collection. Future studies in similar populations could benefit from considering: 1) pre-testing survey instruments within the study population; 2) allowing ample time for staff to develop a rapport with participants; 3) aligning the cultural backgrounds of study staff and participants to facilitate engagement; 4) implementing innovative interventions that that consider food, home, and financial insecurity as well as immigration issues; 5) measuring such insecurity and immigration issues to determine the extent to which they preclude adherence; 6) including ways in which family members can be engaged to positively support behavior change and enhance outcomes; and 7) comprehensive training for those implementing the intervention and collecting data to deal with issues related to social inequality and socio-cultural expectations.

## Data Availability

The datasets analyzed during the current study are available from the corresponding author on reasonable request.

## Abbreviations

*Estudio PARTO*: Project aiming to reduce type two diabetes
GDM: Gestational diabetes mellitus
